# Association between the number of remaining teeth and disability-free life expectancy, and the impact of oral self-care in older Japanese adults: a prospective cohort study

**DOI:** 10.1186/s12877-022-03541-2

**Published:** 2022-10-24

**Authors:** Maya Yamato, Sanae Matsuyama, Yoshitaka Murakami, Jun Aida, Yukai Lu, Yumi Sugawara, Ichiro Tsuji

**Affiliations:** 1 Division of Epidemiology, Department of Health Informatics and Public Health, School of Public Health, Tohoku University Graduate School of Medicine, 2-1, Seiryo-machi, Aoba-ku, Sendai, Miyagi 980-8575 Japan; 2 Department of Medical Statistics, Faculty of Medicine, Toho University, Tokyo, Japan; 3 Department of Oral Health Promotion, Graduate School of Medical and Dental Sciences, Tokyo Medical and Dental University, Tokyo, Japan

**Keywords:** Oral care, Oral health, Healthy life expectancy, Disability-life free expectancy

## Abstract

**Background:**

Tooth loss has been reportedly associated with shorter disability-free life expectancy (DFLE). However, no study has explored whether oral self-care offsets reduction in DFLE. The present study aimed to assess the association between oral self-care and DFLE in older individuals with tooth loss.

**Methods:**

Data on the 13-year follow-up from a cohort study of 14,206 older Japanese adults aged ≥ 65 years in 2006 were analyzed. Information on the number of remaining teeth was collected using a questionnaire, and the participants were then categorized into three groups (0–9, 10–19, and ≥ 20 teeth). Additionally, “0–9” and “10–19” groups were divided into two subgroups based on whether they practiced oral self-care. DFLE was defined as the average number of years a person could expect to live without disability, and was calculated by the multistate life table method based on a Markov model.

**Results:**

DFLE (95% confidence interval) was 19.0 years (18.7–19.4) for 0–9 teeth, 20.1 (19.7–20.5) for 10–19 teeth, and 21.6 (21.2–21.9) for ≥ 20 teeth for men. For women, DFLE was 22.6 (22.3–22.9), 23.5 (23.1–23.8), and 24.7 (24.3–25.1), respectively. Practicing oral self-care was associated with longer DFLE, by 1.6–1.9 years with brushing ≥ 2 times a day in people with 0–9 and 10–19 teeth, and by 3.0–3.1 years with the use of dentures in those with 0–9 teeth.

**Conclusions:**

Practicing oral self-care is associated with an increase in DFLE in older people with tooth loss.

**Supplementary Information:**

The online version contains supplementary material available at 10.1186/s12877-022-03541-2.

## Background

According to a United Nations forecast, the global population aged 60 years and over will triple, from 0.7 billion in 2009 to 2 billion in 2050 [1]. This rapid increase in the older population will lead to a higher prevalence of such chronic conditions as dependence in activities of daily living (ADL) and dementia, thus compromising people’s quality of life (QOL) and increasing the burden on social security. In this context, extending healthy life expectancy (HLE) has become the global public health goal. Previous studies have identified modifiable factors for extending HLE, including blood pressure control, weight control, non-smoking, physical activity, social participation, and so forth [2, 3].

Oral health is a leading candidate for extending HLE, because past studies agreed that there were inverse relations between the number of remaining teeth and mortality risk, incident risks of physical disability, and dementia [4–6]. These findings suggest that a smaller number of remaining teeth would be associated with shorter HLE. To the best of our knowledge, there has been only one study that investigated the association between the number of remaining teeth and HLE. Matsuyama et al. reported that having more remaining teeth was associated with longer HLE in a population of older people in Japan [7]. However, they did not examine the impact of oral self-care on HLE.

We previously reported that practicing oral health care was inversely associated with mortality and disability incidence in older people with tooth loss [8, 9]. In participants with 0–19 teeth, the mortality risk of those practicing oral care decreased by about 46% compared with participants who did not practice oral care [8], and participants with 0–19 teeth without regular dental care had a greater risk of functional disability by approximately 46% than those with ≥ 20 teeth [9].

Because practicing good oral self-care could offset the detrimental effect of shorter DFLE from tooth loss, we can see its benefit in extending HLE. To the best of our knowledge, no study has investigated this association. Clarifying how oral self-care affects HLE would provide an inexpensive method for healthy aging since oral self-care is categorized as primary prevention and can be promoted as population-based prevention.

Therefore, the present study aimed to investigate the association between the number of remaining teeth and the extension of HLE, using a 13-year follow-up of a community-based, large-scale (N = 14,206) cohort study of older Japanese adults. The impact of oral self-care on HLE in those with tooth loss was also examined. Of the various definitions of HLE, the present study focused on disability-free life expectancy (DFLE), which is defined as the average number of years that a person can expect to live without disability because data on the incidence of disability are available from long-term care insurance (LTCI) information.

## Methods

### Study cohort

The details of the Ohsaki Cohort 2006 Study have been introduced previously [10]. In brief, the source population for the baseline survey was all older residents (i.e., 31,694 men and women) living in Ohsaki City, Miyagi Prefecture, Japan aged ≥ 65 years in December 1, 2006 [10]. The questionnaire survey included items on the number of remaining teeth, body weight, height, smoking status, time spent walking, educational status, and history of hypertension, diabetes mellitus, stroke, myocardial infarction, and cancer.

**Fig. 1:**
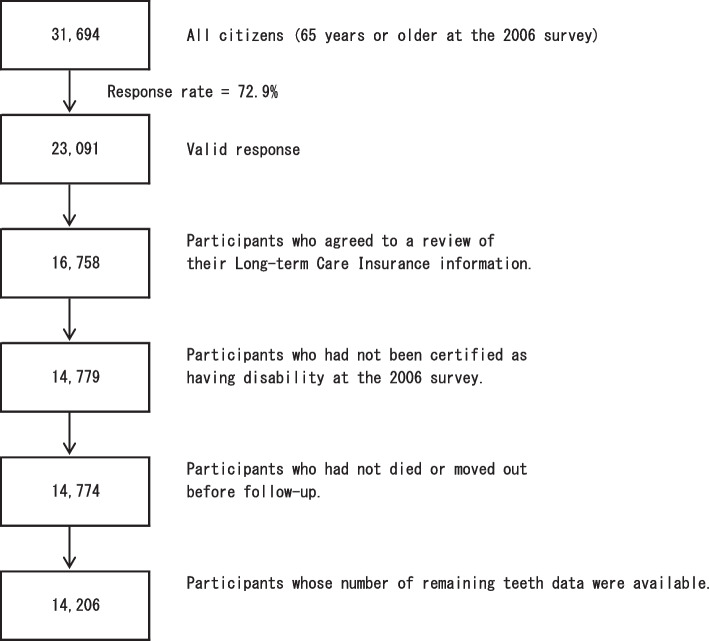
Flowchart of study participants

The baseline survey was conducted between December 1 and 15, 2006, with questionnaires distributed by the heads of individual administrative districts and then collected by mail [10]. Follow-up started from December 16, 2006 until November 30, 2019. For the present analyses, the study cohort consisted of 23,091 participants who provided valid responses. Then 6,333 participants who had not provided written consent for a review of their LTCI information, 1,979 who had already been certified as having a disability by the LTCI (Support Level 1 or higher) before the beginning of follow-up, five who had died or moved before the beginning of follow-up, and 568 whose data about the number of remaining teeth were missing were excluded. The characteristics of those who did not provide written consent for a review of their LTCI information are shown (Supplementary Table 1). Eventually, 14,206 participants were included for the present analyses (Fig. 1).

### Exposure (number of remaining teeth)

In the baseline questionnaire, respondents were asked to classify the number of their remaining teeth into six categories: none (0 teeth), few (1–9 teeth), about half (10–19 teeth), moderate (20–24 teeth), most (25–27 teeth), and all (28 teeth). The respondents were then divided into three groups: (1) 0–9 teeth, (2) 10–19 teeth, and (3) ≥ 20 teeth. Whether they used dentures and whether they visited a dental clinic for dental checkups at least once a year were also asked. The respondents were asked to mark ‘yes’ or ‘no’ in reply. How many times participants brushed their teeth daily was also included as a question.

### Outcomes

The study outcomes were incident disability according to national standards (LTCI Care Level 2 or higher: limited in performing ADL) and death [10]. With these data, DFLE, which was defined as the average number of years that a person could expect to live without disability, was calculated.

LTCI in Japan is a mandatory social insurance system that is meant to help frail older individuals carry out ADL. Everyone aged ≥ 40 years pays a premium, and everyone aged ≥ 65 years is eligible for formal caregiving services depending on the level (Support Level 1–2, and Care Level 1–5). LTCI certification was found to be associated with the ability to perform ADL in a community-based study [11], and it has been used in epidemiologic studies as a measure of incident functional disability in older individuals [12, 13]. Data regarding incident functional disability, death, or emigration during follow-up were transferred from the Ohsaki City Government through an agreement about the secondary use of data. All data were transferred from the Ohsaki City Government yearly each December under the agreement on Epidemiologic Research and Privacy Protection.

### The multistate life table (MSLT) method

The MSLT method was used to analyze HLE [14]. In the present analysis, a Markov transition model for disability and mortality had three states; two non-absorbing states (non-disabled and disabled) and one absorbing state (dead). In the model, four possible health transitions over time were shown as follows: (a) from non-disabled to disabled (the incidence of a disabled status); (b) from disabled to non-disabled (recovery from a disabled status); (c) from non-disabled to dead; and (d) from disabled to dead. In this model, retention status was allowed for the non-disabled and disabled states. (i.e., remaining in disabled status and in disability-free status are another two health transition pathways.) The emigrants after the beginning of the follow-up were included in the calculations.

### Statistical analysis

The DFLE in both non-disabled and disabled states was computed using Interpolated Markov Chain (IMaCh) software (version 0.98r7), which was developed at the Institut national d’études démographiques by Brouard and Lièvre [15]. This well-known software package has been widely used in several recent studies to compute HLE [16–18]. The program has been described in detail in a previous paper [19], so only a brief description is provided here. In the present analysis, a Markov model was created to calculate DFLE. Four age- and group-specific transition probabilities of the Markov model were estimated using multinomial logistic regression. These probabilities were implemented in the MSLT, and the total life expectancy (TLE), DFLE, and disable life expectancy (DLE) were calculated for each subgroup. The participants were categorized into three groups according to the number of remaining teeth. Group-specific DFLEs were then calculated using IMaCh.

Moreover, in this analysis, whether DFLE differed among participants with a fewer number of remaining teeth was examined depending on whether they practiced oral self-care (‘tooth brushing ≥ 2 times per day’, ‘use of dentures’, and ‘taking dental checkups’ being defined as ‘practicing oral self-care’). For this, participants were divided into the following five categories based on three oral self-care measures: (1) ‘non-practicing and having 0–9 teeth’; (2) ‘practicing and having 0–9 teeth’; (3) ‘non-practicing and having 10–19 teeth’; (4) ‘practicing oral self-care and having 10–19 teeth’; and (5) ‘having ≥ 20 teeth’.

Instead of adjusting confounders in the IMaCh program, four stratified analyses were performed, by smoking status (never or former vs. current), body mass index (BMI) (18.5 ≤ BMI < 25.0 vs. BMI < 18.5 or BMI ≥ 25.0 kg/m^2^), time spent walking (≥ 0.5 vs. < 0.5 h/day), and educational status (junior high school or less vs. high school or higher) Furthermore, the effect of oral self-care on DFLE stratified by smoking status, BMI, time spent walking, and educational status were analyzed.

All MSLT methods were performed using the IMaCh software program, and the data preparation and description were made by SAS version 9.4 (SAS Inc., Cary, NC, USA).

## Results

### Participants’ characteristics

A total of 14,206 participants (men: 45.1%) were included in the present analysis, and their mean age (standard deviation) was 73.9 (6.0) years. Only 612 individuals were lost to follow-up because they emigrated from the study area, with a follow-up rate of 95.7%.

Table 1 shows the baseline characteristics according to the number of remaining teeth. Participants in the “0–9 teeth” group were older than other groups, and participants with more remaining teeth were less likely to be female, to be current smokers and to walk < 0.5 h/day, and more likely to practice oral self-care including daily brushing, use of dentures, and regular dental checkup.

**Table 1:** Baseline characteristics of the study participants according to the number of remaining teeth (*n* = 14,206)

	The number of remaining teeth			
	0–9	10–19	≥ 20	*P*-values^a^
No. of subjects	6349	3452	4405	
Age (years) (mean (SD))	76.0 (6.2)	73.1 (5.3)	71.4 (4.9)	< 0.001
Range of Age (years)	65–101	65–94	65–96	
Men (%)	41.7	45.8	49.3	< 0.001
Use of dentures (%)	92.6	75.0	27.6	< 0.001
Brushing ≥ 2 times per day (%)	53.2	62.1	66.5	< 0.001
Regular dental checkup (%)	24.1	52.3	65.1	< 0.001
Body mass index (kg/m2) (mean(SD))	23.3 (3.5)	23.7 (3.3)	23.8 (3.2)	< 0.001
Current smokers (%)	13.0	12.5	9.9	< 0.001
Time spent walking < 0.5 h/d (%)	46.1	33.7	30.7	< 0.001
High school or higher (%)	65.2	71.2	76.4	< 0.001
History of disease (%)				
Hypertension	42.9	43.6	43.4	0.006
Diabetes mellitus	12.6	12.1	10.6	0.334
Stroke	3.1	2.7	2.3	0.043
Myocardial infarction	6.0	4.7	3.9	0.550
Cancer	9.0	8.4	8.6	0.278

^a^Obtained by using *X*^*2*^ test for variables of proportion and one-factor ANOVA for continuous variables (missing value exclude)

At the end of follow-up in 2019, 47.7% were non-disabled, 9.9% were disabled, and 38.1% were dead. Table 2 also shows the sex-specific distribution of outcomes by the number of remaining teeth. Participants with more remaining teeth had a higher proportion of being non-disabled in both men and women.

**Table 2:** The distribution of participants in the outcome in 2019 by the number of remaining teeth (*n* = 14,206)

	The number of remaining teeth							
Outcome	0–9		10–19		≥ 20		Total	
**Men**								
Non-disabled (%)	802	(30.2)	699	(44.2)	1217	(56.0)	2718	(42.4)
Disabled (%)	156	(5.9)	121	(7.6)	147	(6.8)	424	(6.6)
Dead (%)	1586	(59.9)	715	(45.2)	746	(34.2)	3047	(47.6)
Emigrated (%)	105	(4.0)	47	(3.0)	65	(3.0)	217	(3.4)
Total (%)	2649	(100.0)	1582	(100.0)	2175	(100.0)	6406	(100.0)
**Women**								
Non-disabled (%)	1547	(41.8)	1051	(56.2)	1460	(65.5)	4058	(52.0)
Disabled (%)	520	(14.1)	229	(12.2)	233	(10.4)	982	(12.6)
Dead (%)	1439	(38.9)	496	(26.5)	430	(19.3)	2365	(30.3)
Emigrated (%)	194	(5.2)	94	(5.0)	107	(4.8)	395	(5.1)
Total (%)	3700	(100.0)	1870	(100.0)	2230	(100.0)	7800	(100.0)

### Association between the number of remaining teeth and disability-free life expectancy (DFLE)

Table 3 shows DFLE, DLE, and TLE by the number of remaining teeth for men and women at age 65 years. The number of remaining teeth was associated with longer DFLE and TLE for both sexes. DFLE (95% confidence interval) was 19.0 years (18.7–19.4) for “0–9 teeth”, 20.1 (19.7–20.5) for “10–19 teeth”, and 21.6 (21.2–21.9) for “ ≥ 20 teeth” for men, and 22.6 (22.3–22.9), 23.5 (23.1–23.8), and 24.7 (24.3–25.1), respectively, for women. The difference in DFLE between the “0–9 teeth” group and the “ ≥ 20 teeth” group was about 2 years for both sexes (19.0 vs. 21.6 years for men and 22.6 vs. 24.7 years for women).

**Table 3:** DFLE, DLE and TLE at 65 years by the number of remaining teeth (*n* = 14,206)

The number of remaining teeth	The number of participants	DFLE	(95% CI)	DLE	(95% CI)	TLE	(95% CI)
**Men**							
0–9	2649	19.0	(18.7–19.4)	0.9	(0.9–1.0)	19.9	(19.5–20.3)
10–19	1582	20.1	(19.7–20.5)	1.0	(0.9–1.1)	21.1	(20.6–21.5)
≥ 20	2175	21.6	(21.2–21.9)	1.0	(1.0–1.1)	22.6	(22.2–23.0)
**Women**							
0–9	3700	22.6	(22.3–22.9)	3.5	(3.3–3.7)	26.1	(25.7–26.5)
10–19	1870	23.5	(23.1–23.8)	3.8	(3.4–4.2)	27.3	(26.7–27.8)
≥ 20	2230	24.7	(24.3–25.1)	4.3	(3.8–4.8)	29.0	(28.4–29.6)

*DFLE* disability-free life expectancy, *DLE* disabled life expectancy, *TLE* total life expectancy

Table 4 shows DFLE, DLE, and TLE by the number of remaining teeth for men and women at age 65 years by frequency of brushing teeth a day. There was about a 2-year difference in DFLE between those who brushed < 2 times a day and those ≥ 2 times (men: 18.5 vs. 20.1 years; women: 21.7 vs. 23.3 in the “0–9 teeth”, men: 19.3 vs. 21.1 years; women: 22.3 vs. 24.2 in the “10–19 teeth”). In addition, we also compared those who brushed once a day and those ≥ 2 times, differences of DFLE were almost the same as seen in Table 4 (Supplementary Table 2).

**Table 4:** DFLE, DLE, and TLE at 65 years by the number of remaining teeth with brushing

	The number of remaining teeth and daily brushing									
	0–9				10–19				≥ 20	
	< 2 times per day		≥ 2 times per day		< 2 times per day		≥ 2 times per day			
**Men**										
DFLE	18.5	(18.1–19.0)	20.1	(19.5–20.5)	19.3	(18.7–19.8)	21.1	(20.6–21.7)	21.8	(21.4–22.2)
DLE	0.9	(0.9–1.0)	0.9	(0.9–1.0)	1.0	(0.9–1.1)	1.0	(0.9–1.1)	1.1	(1.0–1.1)
TLE	19.4	(19.0–20.0)	21.0	(20.5–21.5)	20.3	(19.6–20.9)	22.1	(21.5–22.7)	22.9	(22.4–23.3)
**Women**										
DFLE	21.7	(21.3–22.1)	23.3	(22.9–23.7)	22.3	(21.7–22.9)	24.2	(23.5–24.8)	24.6	(24.2–25.0)
DLE	3.5	(3.2–3.9)	3.7	(3.3–4.0)	3.8	(3.2–4.4)	4.0	(3.2–4.7)	4.3	(3.8–4.9)
TLE	25.2	(24.7–25.8)	27.0	(26.4–27.5)	26.1	(25.2–26.9)	28.2	(27.4–28.9)	28.9	(28.3–29.6)

*DFLE* disability-free life expectancy, *DLE* disabled life expectancy, *TLE* total life expectancy

Table 5 shows DFLE, DLE, and TLE by the number of remaining teeth for men and women at age 65 years with or without the use of dentures. There was about a 3-year difference in DFLE between those without and with the use of dentures in the “0–9 teeth” group (men: 16.2 vs. 19.3 years; women: 19.8 vs. 22.8). In the “10–19 teeth” group, the difference in DFLE was small (men: 19.5 vs. 20.3 years; women: 22.8 vs. 23.6).

**Table 5:** DFLE, DLE, and TLE at 65 years by the number of remaining teeth with use of dentures

	The number of remaining teeth and use of dentures									
	0–9				10–19				≥ 20	
	Without dentures		With dentures		Without dentures		With dentures			
**Men**										
DFLE	16.2	(15.3–17.1)	19.3	(18.9–19.7)	19.5	(18.8–20.3)	20.3	(19.9–20.8)	21.6	(21.2–22.0)
DLE	0.7	(0.6–0.9)	0.9	(0.9–1.0)	1.0	(0.9–1.1)	1.0	(0.9–1.1)	1.1	(1.0–1.1)
TLE	16.9	(16.0–17.9)	20.2	(19.8–20.6)	20.5	(19.7–21.3)	21.3	(20.8–21.8)	22.7	(22.3–23.1)
**Women**										
DFLE	19.8	(18.9–20.7)	22.8	(22.6–23.2)	22.8	(22.1–23.6)	23.6	(23.2–24.1)	24.7	(24.3–25.1)
DLE	3.4	(2.7–4.1)	3.5	(3.3–3.7)	4.2	(3.3–5.1)	3.7	(3.3–4.2)	4.2	(3.7–4.7)
TLE	23.2	(22.1–24.3)	26.3	(25.9–26.7)	27.0	(26.5–27.6)	27.3	(26.7–28.0)	28.9	(28.2–29.5)

DFLE disability-free life expectancy, DLE disabled life expectancy, TLE total life expectancy

Table 6 shows that there was about 0.5-year difference in DFLE depending on dental checkups (men: 18.9 vs. 19.5 years; women: 22.5 vs. 22.9 in the “0–9 teeth”, men: 19.9 vs. 20.6 years; women: 23.3 vs. 24.0 in the “10–19 teeth”).

**Table 6:** DFLE, DLE, and TLE at 65 years by the number of remaining teeth with dental checkups

	The number of remaining teeth and dental checkups									
	0–9				10–19				≥ 20	
	Without checkups		With checkups		Without checkups		With checkups			
**Men**										
DFLE	18.9	(18.5–19.3)	19.5	(18.9–20.2)	19.9	(19.4–20.4)	20.6	(19.9–21.2)	21.6	(21.2–22.0)
DLE	0.9	(0.8–1.0)	1.0	(0.9–1.1)	1.0	(0.9–1.1)	1.0	(0.9–1.1)	1.1	(1.0–1.1)
TLE	19.8	(19.3–20.2)	20.5	(20.3–21.4)	20.9	(20.3–21.4)	21.6	(20.8–22.3)	22.7	(22.2–23.1)
**Women**										
DFLE	22.5	(22.2–22.8)	22.9	(22.3–23.5)	23.3	(22.8–23.7)	24.0	(23.3–24.6)	24.6	(24.3–25.1)
DLE	3.4	(3.2–3.7)	3.9	(3.3–4.5)	3.7	(3.3–4.2)	4.1	(3.4–4.8)	4.4	(3.9–4.9)
TLE	25.9	(25.5–26.3)	26.8	(26.0–27.7)	27.0	(26.3–27.6)	28.1	(27.1–29.0)	29.0	(28.3–29.5)

*DFLE* disability-free life expectancy, *DLE* disabled life expectancy, *TLE* total life expectancy

### Stratified analysis

Lifestyle factors including smoking status, BMI and time spent walking, and educational status may affect the association between the number of remaining teeth and DFLE. Thus, several stratified analyses were performed by those factors. A consistent association between the number of remaining teeth and DFLE, and an extension of DFLE with daily brushing and use of dentures was also observed, when the participants were stratified by smoking status (Supplementary table 3–5), BMI (Supplementary table 6–8), time spent walking (Supplementary table 9–11), or educational status (Supplementary table 12–14).

## Discussion

In the present study, we estimated DFLEs according to the number of remaining teeth using the data from a cohort study of 14,206 older Japanese men and women aged 65 years or older followed-up for 13 years. DFLE was longer by 2 years in participants with ≥ 20 teeth than those with 0–9 teeth in both men and women. The association between the number of remaining teeth and DFLE did not change greatly after the participants were stratified by smoking status, BMI, time spent walking, and educational status. To our knowledge, this is the first study to investigate the association of the number of remaining teeth and oral self-care with DFLE.

The present result is consistent with the previous study that showed that there was an association between the number of remaining teeth and DFLE [9]. It was further demonstrated that oral self-care may contribute to the extension of DFLE even among older people with tooth loss. Moreover, the difference in DFLE between those with oral self-care and those without it among people with relatively less teeth was also analyzed. When the impact of brushing was investigated, the difference in DFLE between < 2 times/day and ≥ 2 times/day was 1.6 years in the 0–9 teeth group and about 2 years in the 10–19 teeth group. As for the use of dentures, the difference in DFLE between with and without dentures was about 3 years in the 0–9 teeth group and about 1 year in the 10–19 teeth group. From the above, use of dentures seems to alleviate the adverse effect of worse dentition status on DFLE if severe, and it may be more effective for those having much fewer teeth. With respect to dental checkups, the difference in DFLE between with and without checkups was about 0.5 years in both the 0–9 teeth and the 10–19 teeth group. Therefore, promoting oral self-care may extend HLE even when the number of remaining teeth is reduced.

There are several possible pathways linking oral self-care to the extension of DFLE. In this study, use of dentures had a stronger effect on DFLE than those of daily brushing and dental checkups in “0–9” group. Denture can directly affect chewing and swallowing when eating, the support of which may be more fundamental especially in those with severe tooth loss. Tooth brushing would clean up the oral microbiota, which is associated with an increased risk of pneumonia in older people [20]. Possible mechanisms also include the possibility that chewing and swallowing are directly related to cognitive function [21–23]. In addition, denture use would improve chewing and swallowing, thus increasing the amount and kinds of food intake and improving nutritional status [24], eventually preventing sarcopenia [25]. Furthermore, tooth brushing would alleviate oral inflammation, thus decreasing the risk of such systemic diseases as cardiovascular events [26], Alzheimer's disease [27], and obesity [28].

Oral health is a neglected issue on the global health agenda, so it was an important advance when a resolution on oral health was adopted at WHO’s 2021 World Health Assembly [29]. The resolution demands nations to provide a basis for a healthy mouth, where no one is left behind, and to develop “best-buy” interventions for oral health [30]. In the context of this resolution, oral self-care is certainly the “best-buy” option because the present result suggests that it could increase DFLE by about 2 years, preventing the deterioration of QOL including functional disability or onset of disease, and saving tremendous costs of medication and welfare.

The present study had some strengths. First, it was a large population-based cohort study involving 14,206 persons. Second, few participants were lost during follow-up (4.3%).

Some limitations need to be mentioned. First, not all candidates had applied for LTCI certification, and provided consent for a review to their LTCI information. However, we compared baseline characteristics of study participants and those who did not agree to the review to their LTCI information (Supplementary Table 1). Those who agreed to the review to their LTCI information were more likely to be male, to have higher education, but to have medical history of hypertension, myocardial infarction, stroke, and cancer. These results were statistically significant, although the differences were not substantive. Thus, we cannot completely exclude the possibility of selection bias in the present study. Second, information on the number of remaining teeth and dental health behaviors were only assessed once at baseline in the present study, but these variables may change over time. It is also worthwhile to investigate the differences in DFLEs among people with different patterns of changes in oral care habits (e.g., unchanged, decreased, or increased frequency of teeth brushing or dental check-ups), obtained by multiple assessments of variables.

## Conclusions

The results of this study suggest the substantial impact of the number of remaining teeth on longer DFLE among older people. A 2-year difference in DFLE was observed between those who had 0–9 teeth compared with those who had ≥ 20 teeth; this association was consistent for both men and women. In addition, oral self-care could have a great positive impact on the extension of DFLE of those with fewer teeth. Therefore, promoting oral self-care may extend HLE even when the number of remaining teeth is decreased. These findings suggest that maintaining the number of remaining teeth and promoting oral self-care at the population level could increase life-years lived in good health among community-dwelling older people.

## Supplementary Information

**Additional file 1: Supplementary Table 1.** Baseline characteristics according to whether or not agreed to a review of their LTCI information. **Supplementary Table 2.** DFLE, DLE, and TLE at 65 years by the number of remaining teeth with brushing (once vs. twice or more per day). **Supplementary Table 3.** DFLE, DLE, and TLE at 65 years according to the number of remaining teeth stratified by smoking status. **Supplementary Table 4.** DFLE, DLE, and TLE at 65 years according to the number of remaining teeth stratified by daily brushing and smoking status. **Supplementary Table 5.** DFLE, DLE, and TLE at 65 years according to the number of remaining teeth stratified by use of dentures and smoking status. **Supplementary Table 6.** DFLE, DLE, and TLE at 65 years according to the number of teeth stratified by BMI. **Supplementary Table 7.** DFLE, DLE, and TLE at 65 years according to the number of remaining teeth stratified by daily brushing and BMI. **Supplementary Table 8.** DFLE, DLE, and TLE at 65 years according to the number of remaining teeth stratified by use of dentures and BMI. **Supplementary Table 9.** DFLE, DLE, and TLE at 65 years according to the number of teeth stratified by time spent walking. **Supplementary Table 10.** DFLE, DLE, and TLE at 65 years according to the number of remaining teeth stratified by daily brushing and walking. **Supplementary Table 11.** DFLE, DLE, and TLE at 65 years according to the number of remaining teeth stratified by use of dentures and walking. **Supplementary Table 12.** DFLE, DLE, and TLE at 65 years according to the number of remaining teeth stratified by educational status. **Supplementary Table 13.** DFLE, DLE, and TLE at 65 years according to the number of remaining teeth stratified by daily brushing. **Supplementary Table 14.** DFLE, DLE, and TLE at 65 years according to the number of remaining teeth stratified by use of dentures and educational status. 

## Data Availability

Data described in the manuscript, code book, and analytic code will not be made publicly available because private information of participants were included but are available from the corresponding author on reasonable request.

## Abbreviations

HLE: Healthy life expectancy
DFLE: Disability-free life expectancy
ADL: Activities of daily living
TLE: Total life expectancy
DLE: Disabled life expectancy
LTCI: Long-term care insurance
MSLT: Multistate life table
BMI: Body mass index
IMaCh: Interpolated Markov Chain
CI: Confidence interval
QOL: Quality of life
