# Supplementary material for: Association between the number of remaining teeth and disability-free life expectancy, and the impact of oral self-care in older Japanese adults: a prospective cohort study
Source: BMC Geriatr. 2022 Oct 24;22:820. doi: 10.1186/s12877-022-03541-2 (PMC9590145; doi:10.1186/s12877-022-03541-2)
Supplement: Supplementary file 1 — Additional file 1: Supplementary Table 1. Baseline characteristics according to whether or not agreed to a review of their LTCI information. Supplementary Table 2. DFLE, DLE, and TLE at 65 years by the number of remaining teeth with brushing (once vs. twice or more per day). Supplementary Table 3. DFLE, DLE, and TLE at 65 years according to the number of remaining teeth stratified by smoking status. Supplementary Table 4. DFLE, DLE, and TLE at 65 years according to the number of remaining teeth stratified by daily brushing and smoking status. Supplementary Table 5. DFLE, DLE, and TLE at 65 years according to the number of remaining teeth stratified by use of dentures and smoking status. Supplementary Table 6. DFLE, DLE, and TLE at 65 years according to the number of teeth stratified by BMI. Supplementary Table 7. DFLE, DLE, and TLE at 65 years according to the number of remaining teeth stratified by daily brushing and BMI. Supplementary Table 8. DFLE, DLE, and TLE at 65 years according to the number of remaining teeth stratified by use of dentures and BMI. Supplementary Table 9. DFLE, DLE, and TLE at 65 years according to the number of teeth stratified by time spent walking. Supplementary Table 10. DFLE, DLE, and TLE at 65 years according to the number of remaining teeth stratified by daily brushing and walking. Supplementary Table 11. DFLE, DLE, and TLE at 65 years according to the number of remaining teeth stratified by use of dentures and walking. Supplementary Table 12. DFLE, DLE, and TLE at 65 years according to the number of remaining teeth stratified by educational status. Supplementary Table 13. DFLE, DLE, and TLE at 65 years according to the number of remaining teeth stratified by daily brushing. Supplementary Table 14. DFLE, DLE, and TLE at 65 years according to the number of remaining teeth stratified by use of dentures and educational status. [file 12877_2022_3541_MOESM1_ESM.docx]

Supplementary Table 1. Baseline characteristics according to whether or not agreed to a review of their LTCI information.

|  | Participants who did not agree to a  review to their LTCI information | Participants who agreed to a  review to their LTCI information | | *P*-values^a^ |
| --- | --- | --- | --- | --- |
| No. of subjects | 6333 | 16758 | |  |
| Age (years) (mean (SD)) | 74.9 (6.5) | 74.9 (6.6) | | 0.756 |
| Men (%) | 41.7 | 45.8 | | <0.001 |
| Use of dentures (%) | 31.3 | 30.5 | | 0.267 |
| Brushing ≥2 times per day (%) | 56.7 | 57.9 | | 0.113 |
| Regular dental checkup (%) | 72.8 | 71.9 | | 0.237 |
| Body mass index (kg/m2) (mean(SD)) | 23.5 (3.5) | 23.5 (3.5) | | 0.377 |
| Current smokers (%) | 13.0 | 12.5 | | <0.001 |
| Time spent walking <0.5 h/d (%) | 40.3 | 40.3 | | 0.97 |
| High school or higher (%) | 60.3 | 68.4 | | <0.001 |
| History of disease (%) |  |  | |  |
| Hypertension | 39.4 | 43.4 | | <0.001 |
| Diabetes mellitus | 11.4 | 12.2 | | 0.120 |
| Stroke | 3.7 | 4.8 | | <0.001 |
| Myocardial infarction | 4.5 | 5.5 | | <0.001 |
| Cancer | 6.2 | 8.8 | | <0.001 |
| ^a^Obtained by using *X^2^* test for variables of proportion and Student’s T-test for continuous variables (missing value exclude). | | |  |  |

Supplementary Table 2. DFLE, DLE, and TLE at 65 years by the number of remaining teeth with brushing (once vs. twice or more per day).

|  | The number of remaining teeth and daily brushing | | | | | | | | | | | | | |
| --- | --- | --- | --- | --- | --- | --- | --- | --- | --- | --- | --- | --- | --- | --- |
|  | 0–9 | | | | |  | 10–19 | | | | |  | ≥20 | |
|  | once per day | |  | Twice or more  per day | |  | once per day | |  | Twice or more  per day | |  |  |  |
| **Men** |  | |  |  | |  |  | |  |  | |  |  | |
| DFLE | 18.7 | (18.3–19.1) |  | 20.0 | (19.5–20.5) |  | 19.3 | (18.7–19.9) |  | 21.1 | (20.5–21.7) |  | 21.8 | (21.4–22.2) |
| DLE | 1.0 | (0.9–1.0) |  | 0.9 | (0.9–1.0) |  | 1.0 | (0.9–1.1) |  | 1.0 | (0.9–1.1) |  | 1.1 | (1.0–1.1) |
| TLE | 19.7 | (19.0–20.0) |  | 21.0 | (20.5–21.5) |  | 20.3 | (19.8–20.8) |  | 22.1 | (21.5–22.7) |  | 22.9 | (22.5–23.3) |
| **Women** |  |  |  |  |  |  |  |  |  |  |  |  |  |  |
| DFLE | 21.9 | (21.5–22.3) |  | 23.3 | (22.9–23.7) |  | 22.3 | (21.7–22.9) |  | 24.2 | (23.7–24.7) |  | 24.6 | (24.2–25.0) |
| DLE | 3.5 | (3.2–3.8) |  | 3.7 | (3.3–4.0) |  | 3.8 | (3.2–4.4) |  | 4.0 | (3.5–4.5) |  | 4.3 | (3.8–4.9) |
| TLE | 25.5 | (25.0–26.0) |  | 27.0 | (26.5–27.5) |  | 26.1 | (25.3–26.9) |  | 28.2 | (27.4–28.9) |  | 28.9 | (28.3–29.6) |

Supplementary Table 3. DFLE, DLE, and TLE at 65 years according to the number of remaining teeth stratified by smoking status.

| Number of remaining teeth | DFLE | (95% CI) | DLE | (95% CI) | TLE | (95% CI) |
| --- | --- | --- | --- | --- | --- | --- |
| **Men** |  |  |  |  |  |  |
| **Never or former smokers** |  |  |  |  |  |  |
| 0–9 | 19.8 | (19.4–20.2) | 1.0 | (0.9–1.0) | 20.8 | (20.3–21.2) |
| 10–19 | 20.9 | (20.4–21.3) | 1.0 | (1.0–1.1) | 21.9 | (21.4–22.4) |
| ≥20 | 22.1 | (21.7–22.5) | 1.1 | (1.0–1.2) | 23.2 | (22.8–23.7) |
| **Current smokers** |  |  |  |  |  |  |
| 0–9 | 17.2 | (16.7–17.7) | 0.8 | (0.7–0.9) | 18.0 | (17.4–18.5) |
| 10–19 | 18.2 | (17.7–18.8) | 0.9 | (0.8–1.0) | 19.1 | (18.5–19.7) |
| ≥20 | 19.5 | (18.9–20.1) | 0.9 | (0.8–1.0) | 20.4 | (19.8–21.0) |
| **Women** |  |  |  |  |  |  |
| **Never or former smokers** |  |  |  |  |  |  |
| 0–9 | 22.6 | (22.3–23.0) | 3.5 | (3.3–3.7) | 26.1 | (25.7–26.5) |
| 10–19 | 23.5 | (23.0–23.9) | 3.9 | (3.4–4.3) | 27.4 | (26.7–28.0) |
| ≥20 | 24.5 | (24.1–25.0) | 4.3 | (3.7–4.8) | 28.8 | (28.1–29.5) |
| **Current smokers** |  |  |  |  |  |  |
| 0–9 | 20.2 | (19.6–20.8) | 2.8 | (2.2–3.5) | 23.0 | (22.2–23.9) |
| 10–19 | 21.2 | (20.4–21.8) | 3.1 | (2.3–4.0) | 24.3 | (23.3–25.3) |
| ≥20 | 22.2 | (21.5–22.9) | 3.5 | (2.5–4.4) | 25.7 | (24.6–26.8) |

| Number of remaining teeth | Daily brushing | DFLE | (95% CI) | DLE | (95% CI) | TLE | (95% CI) |
| --- | --- | --- | --- | --- | --- | --- | --- |
| **Men** |  |  |  |  |  |  |  |
| **Never or former smokers** |  |  |  |  |  |  |  |
| 0–9 | <2 times per day | 19.3 | (18.8–19.8) | 1.0 | (0.9–1.1) | 20.3 | (19.8–20.8) |
|  | ≥2 times per day | 20.7 | (20.2–21.2) | 0.9 | (0.8–1.0) | 21.6 | (21.0–22.2) |
| 10–19 | <2 times per day | 19.9 | (19.3–20.5) | 1.0 | (0.9–1.1) | 20.9 | (20.2–21.6) |
|  | ≥2 times per day | 21.9 | (21.3–22.5) | 1.0 | (0.9–1.1) | 22.9 | (22.2–23.6) |
| **Current smokers** |  |  |  |  |  |  |  |
| 0–9 | <2 times per day | 16.8 | (16.2–17.4) | 0.8 | (1.0–1.6) | 17.6 | (17.0–18.2) |
|  | ≥2 times per day | 18.1 | (17.5–18.7) | 0.8 | (0.7–0.9) | 18.9 | (18.2–19.6) |
| 10–19 | <2 times per day | 17.4 | (16.7–18.1) | 0.8 | (0.7–0.9) | 18.2 | (17.4–19.0) |
|  | ≥2 times per day | 19.3 | (18.5–20.1) | 0.8 | (0.7–0.9) | 20.1 | (19.3–20.9) |
| **Women** |  |  |  |  |  |  |  |
| **Never or former smokers** |  |  |  |  |  |  |  |
| 0–9 | <2 times per day | 21.7 | (21.2–22.2) | 3.7 | (3.3–4.1) | 25.4 | (24.8–26.0) |
|  | ≥2 times per day | 23.4 | (23.0–23.8) | 3.6 | (3.3–3.9) | 26.9 | (26.3–27.5) |
| 10–19 | <2 times per day | 22.2 | (21.5–22.9) | 3.8 | (3.1–4.5) | 26.0 | (25.1–26.9) |
|  | ≥2 times per day | 24.2 | (23.6–24.8) | 3.9 | (3.3–4.5) | 28.1 | (27.3–28.9) |
| **Current smokers** |  |  |  |  |  |  |  |
| 0–9 | <2 times per day | 19.5 | (18.7–20.3) | 2.9 | (2.1–3.7) | 22.5 | (21.5–23.5) |
|  | ≥2 times per day | 21.1 | (20.3–21.9) | 2.8 | (2.0–3.6) | 23.9 | (22.8–25.0) |
| 10–19 | <2 times per day | 20.1 | (19.2–21.0) | 3.1 | (2.1–4.1) | 23.2 | (22.0–24.4) |
|  | ≥2 times per day | 22.0 | (21.1–22.9) | 3.1 | (2.1–4.1) | 25.2 | (24.0–26.4) |

Supplementary Table 4. DFLE, DLE, and TLE at 65 years according to the number of remaining teeth stratified by daily brushing and smoking status.

| Number of remaining teeth | Use of dentures | DFLE | (95% CI) | DLE | (95% CI) | TLE | (95% CI) |
| --- | --- | --- | --- | --- | --- | --- | --- |
| **Men** |  |  |  |  |  |  |  |
| **Never or former smokers** |  |  |  |  |  |  |  |
| 0–9 | No | 16.9 | (15.9–17.9) | 0.8 | (0.7–0.9) | 17.7 | (16.7–18.7) |
|  | Yes | 20.1 | (19.7–20.5) | 1.0 | (0.9–1.1) | 21.1 | (20.6–21.6) |
| 10–19 | No | 20.4 | (19.6–21.2) | 1.0 | (0.9–1.1) | 21.4 | (20.5–22.3) |
|  | Yes | 21.1 | (19.5–21.5) | 1.0 | (0.9–1.1) | 22.1 | (21.6–22.6) |
| **Current smokers** |  |  |  |  |  |  |  |
| 0–9 | No | 14.4 | (13.4–15.4) | 0.6 | (0.5–0.7) | 15.0 | (14.0–16.0) |
|  | Yes | 17.4 | (16.8–18.0) | 0.8 | (0.7–0.9) | 18.2 | (17.6–18.8) |
| 10–19 | No | 17.8 | (16.9–18.7) | 0.8 | (0.7–0.9) | 18.6 | (17.6–19.6) |
|  | Yes | 18.4 | (17.7–19.1) | 0.8 | (0.7–0.9) | 19.2 | (18.5–19.9) |
| **Women** |  |  |  |  |  |  |  |
| **Never or former smokers** |  |  |  |  |  |  |  |
| 0–9 | No | 19.9 | (18.9–20.9) | 3.4 | (2.6–4.2) | 23.3 | (22.1–24.4) |
|  | Yes | 22.9 | (22.5–23.4) | 3.5 | (3.3–3.7) | 26.4 | (26.0–26.8) |
| 10–19 | No | 23.1 | (22.3–23.9) | 4.1 | (3.1–5.1) | 27.2 | (26.0–28.4) |
|  | Yes | 23.7 | (23.2–24.2) | 3.7 | (3.2–4.2) | 27.4 | (26.7–28.1) |
| **Current smokers** |  |  |  |  |  |  |  |
| 0–9 | No | 17.6 | (16.5–18.7) | 2.5 | (1.6–3.4) | 20.1 | (18.8–21.4) |
|  | Yes | 20.6 | (19.9–21.3) | 2.7 | (2.0–3.4) | 23.3 | (22.8–23.8) |
| 10–19 | No | 20.9 | (20.4–21.4) | 3.1 | (2.5–3.7) | 24.0 | (22.6–25.4) |
|  | Yes | 21.4 | (20.6–22.2) | 2.9 | (2.1–3.7) | 24.3 | (23.2–25.6) |

Supplementary Table 5. DFLE, DLE, and TLE at 65 years according to the number of remaining teeth stratified by use of dentures and smoking status.

Supplementary Table 6. DFLE, DLE, and TLE at 65 years according to the number of teeth stratified by BMI.

| Number of remaining teeth | DFLE | (95% CI) | DLE | (95% CI) | TLE | (95% CI) |
| --- | --- | --- | --- | --- | --- | --- |
| **Men** |  |  |  |  |  |  |
| **18.5≤ BMI <25.0** |  |  |  |  |  |  |
| 0–9 | 19.1 | (18.6–19.6) | 0.9 | (0.8–1.0) | 20.0 | (19.5–20.5) |
| 10–19 | 20.1 | (19.6–20.6) | 1.0 | (0.9–1.1) | 21.1 | (20.5–21.5) |
| ≥20 | 21.7 | (20.9–22.2) | 1.0 | (0.9–1.1) | 22.7 | (20.4–21.6) |
| **BMI<18.5 or 25.0≤ BMI** |  |  |  |  |  |  |
| 0–9 | 19.3 | (18.9–19.7) | 0.9 | (0.8–1.0) | 20.3 | (19.8–20.6) |
| 10–19 | 20.2 | (19.8–20.8) | 1.0 | (0.9–1.1) | 21.2 | (20.7–21.7) |
| ≥20 | 21.8 | (21.4–22.2) | 1.1 | (1.0–1.2) | 22.9 | (22.5–23.5) |
| **Women** |  |  |  |  |  |  |
| **18.5≤ BMI <25.0** |  |  |  |  |  |  |
| 0–9 | 22.8 | (22.4–23.2) | 3.5 | (3.2–3.8) | 26.3 | (25.9–26.9) |
| 10–19 | 23.6 | (23.1–24.1) | 3.9 | (3.4–4.4) | 27.5 | (28.8–30.9) |
| ≥20 | 24.9 | (24.8–26.1) | 4.4 | (3.5–5.5) | 29.3 | (28.7–29.8) |
| **BMI<18.5 or 25.0≤ BMI** |  |  |  |  |  |  |
| 0–9 | 22.9 | (22.5–23.3) | 3.5 | (0.8–1.0) | 26.4 | (25.9–26.9) |
| 10–19 | 23.6 | (23.1–24.1) | 3.8 | (3.2–3.7) | 27.4 | (26.8–28.0) |
| ≥20 | 24.9 | (24.4–25.4) | 4.3 | (3.7–4.9) | 29.2 | (28.5–29.9) |

Supplementary Table 7. DFLE, DLE, and TLE at 65 years according to the number of remaining teeth stratified by daily brushing and BMI.

| Number of remaining teeth | Daily brushing | DFLE | (95% CI) | DLE | (95% CI) | TLE | (95% CI) |
| --- | --- | --- | --- | --- | --- | --- | --- |
| **Men** |  |  |  |  |  |  |  |
| **18.5≤ BMI <25.0** |  |  |  |  |  |  |  |
| 0–9 | <2 times per day | 18.8 | (18.3–19.3) | 0.9 | (0.8–1.0) | 19.7 | (19.1–20.3) |
|  | ≥2 times per day | 20.3 | (19.7–20.9) | 0.9 | (0.8–1.0) | 21.2 | (20.6–21.8) |
| 10–19 | <2 times per day | 19.4 | (18.7–20.1) | 1.0 | (0.9–1.1) | 20.4 | (19.7–21.1) |
|  | ≥2 times per day | 21.2 | (20.6–21.8) | 1.0 | (0.9–1.1) | 22.2 | (21.5–22.9) |
| **BMI<18.5 or 25.0≤ BMI** |  |  |  |  |  |  |  |
| 0–9 | <2 times per day | 18.7 | (18.1–19.3) | 0.9 | (0.8–1.0) | 19.6 | (19.0–20.2) |
|  | ≥2 times per day | 20.2 | (19.5–20.9) | 0.9 | (0.8–1.0) | 21.1 | (20.4–21.8) |
| 10–19 | <2 times per day | 19.3 | (18.6–20.0) | 1.0 | (0.9–1.1) | 20.3 | (19.5–21.1) |
|  | ≥2 times per day | 21.1 | (20.4–21.8) | 1.0 | (0.9–1.1) | 22.1 | (21.3–22.9) |
| **Women** |  |  |  |  |  |  |  |
| **18.5≤ BMI <25.0** |  |  |  |  |  |  |  |
| 0–9 | <2 times per day | 22.0 | (21.5–22.5) | 3.5 | (3.1–3.9) | 25.5 | (24.8–26.2) |
|  | ≥2 times per day | 23.6 | (23.1–24.1) | 3.6 | (3.2–4.0) | 27.2 | (26.6–27.8) |
| 10–19 | <2 times per day | 22.5 | (21.8–23.2) | 3.6 | (2.9–4.3) | 26.1 | (25.2–27.0) |
|  | ≥2 times per day | 24.3 | (23.7–24.9) | 3.9 | (3.3–4.5) | 28.2 | (27.4–29.0) |
| **BMI<18.5 or 25.0≤ BMI** |  |  |  |  |  |  |  |
| 0–9 | <2 times per day | 21.9 | (21.3–22.5) | 3.5 | (3.1–3.9) | 25.4 | (24.7–26.1) |
|  | ≥2 times per day | 23.5 | (23.0–24.0) | 3.6 | (3.1–4.1) | 27.1 | (26.4–27.8) |
| 10–19 | <2 times per day | 22.4 | (21.7–23.1) | 3.6 | (2.9–4.3) | 26.0 | (25.0–27.0) |
|  | ≥2 times per day | 24.3 | (23.7–24.9) | 3.9 | (3.2–4.6) | 28.2 | (27.2–29.2) |

Supplementary Table 8. DFLE, DLE, and TLE at 65 years according to the number of remaining teeth stratified by use of dentures and BMI.

| Number of remaining teeth | Use of dentures | DFLE | (95% CI) | DLE | (95% CI) | TLE | (95% CI) |
| --- | --- | --- | --- | --- | --- | --- | --- |
| **Men** |  |  |  |  |  |  |  |
| **18.5≤ BMI <25.0** |  |  |  |  |  |  |  |
| 0–9 | No | 16.3 | (15.3–17.3) | 0.7 | (0.6–0.8) | 17.0 | (15.9–18.1) |
|  | Yes | 19.5 | (19.1–19.9) | 0.9 | (0.8–1.0) | 20.4 | (19.9–20.9) |
| 10–19 | No | 19.6 | (18.8–20.4) | 0.9 | (0.8–1.0) | 20.5 | (19.6–21.4) |
|  | Yes | 20.4 | (19.9–20.9) | 1.0 | (0.9–1.1) | 21.4 | (20.8–22.0) |
| **BMI<18.5 or 25.0≤ BMI** |  |  |  |  |  |  |  |
| 0–9 | No | 16.1 | (15.1–17.1) | 0.7 | (0.6–0.8) | 16.8 | (15.7–17.9) |
|  | Yes | 19.3 | (18.8–19.8) | 0.9 | (0.8–1.0) | 20.2 | (19.6–20.8) |
| 10–19 | No | 19.5 | (18.6–20.4) | 0.9 | (0.8–1.0) | 20.4 | (19.4–21.4) |
|  | Yes | 20.3 | (19.7–20.9) | 1.0 | (0.9–1.1) | 21.3 | (20.6–22.0) |
| **Women** |  |  |  |  |  |  |  |
| **18.5≤ BMI <25.0** |  |  |  |  |  |  |  |
| 0–9 | No | 20.3 | (19.3–21.3) | 2.9 | (2.2–3.6) | 23.2 | (22.0–24.4) |
|  | Yes | 23.2 | (22.8–23.6) | 3.5 | (3.2–3.8) | 26.7 | (26.2–27.2) |
| 10–19 | No | 23.2 | (22.4–24.0) | 3.9 | (3.0–4.8) | 27.1 | (25.9–28.3) |
|  | Yes | 23.9 | (23.4–24.4) | 3.7 | (3.2–4.2) | 27.6 | (26.9–28.3) |
| **BMI<18.5 or 25.0≤ BMI** |  |  |  |  |  |  |  |
| 0–9 | No | 20.1 | (19.1–21.1) | 2.9 | (2.1–3.7) | 23.0 | (21.7–24.3) |
|  | Yes | 23.1 | (22.6–23.6) | 3.6 | (3.2–4.0) | 26.7 | (26.1–27.3) |
| 10–19 | No | 23.2 | (22.4–24.0) | 3.9 | (3.0–4.8) | 27.1 | (25.9–28.3) |
|  | Yes | 23.9 | (23.4–24.4) | 3.7 | (3.2–4.2) | 27.6 | (26.9–28.3) |

Supplementary Table 9. DFLE, DLE, and TLE at 65 years according to the number of teeth stratified by time spent walking.

| Number of remaining teeth | DFLE | (95% CI) | DLE | (95% CI) | TLE | (95% CI) |
| --- | --- | --- | --- | --- | --- | --- |
| **Men** |  |  |  |  |  |  |
| **<0.5 hours/day** |  |  |  |  |  |  |
| 0–9 | 17.5 | (17.0–17.9) | 1.3 | (1.2–1.4) | 18.8 | (18.3–19.3) |
| 10–19 | 18.4 | (17.9–18.9) | 1.5 | (1.4–1.7) | 19.9 | (19.3–20.4) |
| ≥20 | 19.8 | (19.4–20.3) | 1.8 | (1.6–2.0) | 21.6 | (21.1–22.2) |
| **≥0.5 hours/day** |  |  |  |  |  |  |
| 0–9 | 19.8 | (19.4–20.1) | 1.4 | (1.3–1.5) | 21.2 | (20.7–21.6) |
| 10–19 | 20.7 | (20.2–21.1) | 1.6 | (1.5–1.8) | 22.3 | (21.8–22.8) |
| ≥20 | 22.2 | (21.8–22.6) | 1.9 | (1.7–2.1) | 24.1 | (23.6–24.6) |
| **Women** |  |  |  |  |  |  |
| **<0.5 hours/day** |  |  |  |  |  |  |
| 0–9 | 21.4 | (21.1–21.8) | 3.4 | (3.1–3.6) | 24.8 | (24.6–25.3) |
| 10–19 | 22.2 | (21.8–22.6) | 3.9 | (3.5–4.3) | 26.1 | (25.4–26.7) |
| ≥20 | 23.4 | (22.9–23.8) | 4.6 | (4.1–5.1) | 28.0 | (27.2–28.6) |
| **≥0.5 hours/day** |  |  |  |  |  |  |
| 0–9 | 23.7 | (23.2–24.1) | 3.6 | (3.2–4.0) | 27.3 | (26.6–27.9) |
| 10–19 | 24.3 | (23.9–24.8) | 4.2 | (3.7–4.6) | 28.5 | (27.9–29.1) |
| ≥20 | 25.6 | (25.1–26.0) | 4.8 | (4.3–5.5) | 30.4 | (29.7–31.2) |

Supplementary Table 10. DFLE, DLE, and TLE at 65 years according to the number of remaining teeth stratified by daily brushing and walking.

| Number of remaining teeth | Daily brushing | DFLE | (95% CI) | DLE | (95% CI) | TLE | (95% CI) |
| --- | --- | --- | --- | --- | --- | --- | --- |
| **Men** |  |  |  |  |  |  |  |
| **<0.5 hours/day** |  |  |  |  |  |  |  |
| 0–9 | <2 times per day | 17.1 | (16.6–17.6) | 0.9 | (0.8–1.0) | 18.0 | (17.4–18.6) |
|  | ≥2 times per day | 18.4 | (17.8–19.0) | 0.9 | (0.8–1.0) | 19.3 | (18.7–19.9) |
| 10–19 | <2 times per day | 17.6 | (16.9–18.3) | 1.0 | (0.9–1.1) | 18.6 | (17.9–19.3) |
|  | ≥2 times per day | 19.4 | (18.7–20.1) | 1.0 | (0.9–1.1) | 20.4 | (19.7–21.1) |
| **≥0.5 hours/day** |  |  |  |  |  |  |  |
| 0–9 | <2 times per day | 19.4 | (18.9–19.9) | 1.0 | (0.9–1.1) | 20.4 | (19.9–20.9) |
|  | ≥2 times per day | 20.8 | (20.3–21.3) | 0.9 | (0.8–1.0) | 21.7 | (21.1–22.3) |
| 10–19 | <2 times per day | 20.0 | (19.4–20.6) | 1.0 | (0.9–1.1) | 21.0 | (19.3–21.7) |
|  | ≥2 times per day | 21.8 | (21.2–22.4) | 1.0 | (0.9–1.1) | 22.8 | (22.1–23.5) |
| **Women** |  |  |  |  |  |  |  |
| **<0.5 hours/day** |  |  |  |  |  |  |  |
| 0–9 | <2 times per day | 20.6 | (20.1–21.1) | 3.5 | (3.1–3.9) | 24.1 | (23.5–24.7) |
|  | ≥2 times per day | 22.1 | (21.6–22.6) | 3.6 | (3.2–4.0) | 25.7 | (25.1–26.3) |
| 10–19 | <2 times per day | 21.0 | (20.3–21.7) | 3.7 | (3.1–4.3) | 24.7 | (23.8–25.6) |
|  | ≥2 times per day | 22.9 | (22.3–23.5) | 3.8 | (3.2–4.2) | 26.7 | (25.9–27.5) |
| **≥0.5 hours/day** |  |  |  |  |  |  |  |
| 0–9 | <2 times per day | 22.8 | (22.3–23.3) | 3.6 | (3.2–4.0) | 26.4 | (25.8–27.0) |
|  | ≥2 times per day | 24.4 | (23.9–24.9) | 3.7 | (3.3–4.1) | 28.1 | (27.5–28.7) |
| 10–19 | <2 times per day | 23.2 | (22.5–23.9) | 3.8 | (3.2–4.4) | 27.1 | (26.2–28.0) |
|  | ≥2 times per day | 25.2 | (24.6–25.8) | 3.9 | (3.3–4.5) | 29.1 | (28.3–29.9) |

Supplementary Table 11. DFLE, DLE, and TLE at 65 years according to the number of remaining teeth stratified by use of dentures and walking.

| Number of remaining teeth | Use of dentures | DFLE | (95% CI) | DLE | (95% CI) | TLE | (95% CI) |
| --- | --- | --- | --- | --- | --- | --- | --- |
| **Men** |  |  |  |  |  |  |  |
| **<0.5 hours/day** |  |  |  |  |  |  |  |
| 0–9 | No | 14.9 | (14.0–15.8) | 0.7 | (0.6–0.8) | 15.6 | (14.6–16.6) |
|  | Yes | 17.7 | (17.2–18.2) | 0.9 | (0.8–1.0) | 18.6 | (18.1–19.1) |
| 10–19 | No | 18.0 | (17.2–18.8) | 0.9 | (0.8–1.0) | 18.9 | (18.0–19.8) |
|  | Yes | 18.6 | (18.3–19.2) | 1.0 | (0.9–1.1) | 19.6 | (19.0–20.2) |
| **≥0.5 hours/day** |  |  |  |  |  |  |  |
| 0–9 | No | 17.0 | (16.1–17.9) | 0.8 | (0.7–0.9) | 17.8 | (16.8–18.8) |
|  | Yes | 20.0 | (19.6–20.4) | 0.9 | (0.8–1.0) | 20.9 | (20.5–21.5) |
| 10–19 | No | 20.3 | (19.5–21.1) | 1.0 | (0.9–1.1) | 21.3 | (20.4–22.2) |
|  | Yes | 21.0 | (20.5–21.5) | 1.0 | (0.9–1.1) | 22.0 | (21.5–22.5) |
| **Women** |  |  |  |  |  |  |  |
| **<0.5 hours/day** |  |  |  |  |  |  |  |
| 0–9 | No | 18.8 | (18.3–19.3) | 3.5 | (2.7–4.3) | 22.3 | (21.1–23.5) |
|  | Yes | 21.7 | (21.3–22.1) | 3.4 | (3.1–3.7) | 25.1 | (24.6–25.6) |
| 10–19 | No | 21.8 | (21.4–22.4) | 3.9 | (3.1–4.7) | 25.7 | (24.6–26.8) |
|  | Yes | 22.4 | (21.9–22.9) | 3.7 | (3.2–4.2) | 26.1 | (25.4–26.8) |
| **≥0.5 hours/day** |  |  |  |  |  |  |  |
| 0–9 | No | 21.0 | (20.0–22.0) | 3.7 | (2.9–4.5) | 24.7 | (23.5–25.9) |
|  | Yes | 23.9 | (23.5–24.3) | 3.6 | (3.3–3.9) | 27.5 | (27.0–28.0) |
| 10–19 | No | 24.0 | (23.2–24.8) | 4.1 | (3.2–5.0) | 28.1 | (26.9–29.3) |
|  | Yes | 24.6 | (24.1–25.1) | 3.8 | (3.3–4.3) | 28.4 | (27.7–29.1) |

Supplementary Table 12. DFLE, DLE, and TLE at 65 years according to the number of remaining teeth stratified by educational status.

| Number of remaining teeth | DFLE | (95% CI) | DLE | (95% CI) | TLE | (95% CI) |
| --- | --- | --- | --- | --- | --- | --- |
| **Men** |  |  |  |  |  |  |
| **Junior high school or less** |  |  |  |  |  |  |
| 0–9 | 18.4 | (18.0–18.8) | 1.4 | (1.3–1.5) | 19.8 | (19.3–20.3) |
| 10–19 | 19.3 | (18.8–19.8) | 1.7 | (1.5–1.7) | 21.0 | (20.4–21.6) |
| ≥20 | 20.8 | (20.3–21.3) | 1.9 | (1.7–2.1) | 22.7 | (22.6–23.6) |
| **High school or higher** |  |  |  |  |  |  |
| 0–9 | 19.5 | (19.1–19.9) | 1.3 | (0.7–0.9) | 20.8 | (20.4–21.2) |
| 10–19 | 20.5 | (20.1–20.9) | 1.6 | (0.8–1.0) | 22.1 | (21.6–22.6) |
| ≥20 | 22.0 | (21.6–22.4) | 1.8 | (0.8–1.0) | 23.8 | (23.3–24.3) |
| **Women** |  |  |  |  |  |  |
| **Junior high school or less** |  |  |  |  |  |  |
| 0–9 | 21.9 | (21.5–22.3) | 3.7 | (3.4–4.0) | 25.6 | (25.1–26.1) |
| 10–19 | 22.7 | (22.3–23.1) | 4.4 | (3.9–4.9) | 27.1 | (26.3–27.7) |
| ≥20 | 23.8 | (23.3–24.3) | 4.9 | (4.3–5.5) | 28.7 | (27.9–29.5) |
| **High school or higher** |  |  |  |  |  |  |
| 0–9 | 23.1 | (22.8–23.4) | 3.4 | (3.2–3.6) | 26.5 | (26.2–27.0) |
| 10–19 | 23.9 | (23.5–24.3) | 4.1 | (3.7–4.5) | 28.0 | (27.4–28.6) |
| ≥20 | 25.1 | (24.7–25.5) | 4.6 | (4.1–5.1) | 29.7 | (29.0–30.4) |

| Number of remaining teeth | Daily brushing | DFLE | (95% CI) | DLE | (95% CI) | TLE | (95% CI) |
| --- | --- | --- | --- | --- | --- | --- | --- |
| **Men** |  |  |  |  |  |  |  |
| **Junior high school or less** |  |  |  |  |  |  |  |
| 0–9 | <2 times per day | 18.0 | (17.5–18.5) | 1.5 | (1.3–1.7) | 19.5 | (18.9–20.1) |
|  | ≥2 times per day | 19.5 | (18.9–20.1) | 1.5 | (1.3–1.7) | 21.0 | (20.3–21.7) |
| 10–19 | <2 times per day | 18.7 | (18.0–19.4) | 1.7 | (1.5–1.9) | 20.4 | (19.6–21.2) |
|  | ≥2 times per day | 20.4 | (19.7–21.1) | 1.7 | (1.5–1.9) | 22.1 | (21.3–22.9) |
| **High school or higher** |  |  |  |  |  |  |  |
| 0–9 | <2 times per day | 18.9 | (18.4–19.4) | 1.4 | (1.3–1.5) | 20.3 | (19.7–20.9) |
|  | ≥2 times per day | 20.4 | (19.9–20.9) | 1.4 | (1.3–1.5) | 21.8 | (21.2–22.4) |
| 10–19 | <2 times per day | 19.6 | (18.8–20.4) | 1.5 | (1.3–1.7) | 21.1 | (19.3–21.7) |
|  | ≥2 times per day | 21.8 | (21.2–22.4) | 1.0 | (0.9–1.1) | 22.8 | (22.1–23.5) |
| **Women** |  |  |  |  |  |  |  |
| **Junior high school or less** |  |  |  |  |  |  |  |
| 0–9 | <2 times per day | 21.1 | (20.5–21.7) | 3.8 | (3.0–4.6) | 24.9 | (24.2–25.6) |
|  | ≥2 times per day | 22.8 | (22.3–23.3) | 3.8 | (3.4–4.2) | 26.6 | (25.9–27.3) |
| 10–19 | <2 times per day | 21.7 | (21.0–22.4) | 4.3 | (3.6–5.0) | 26.0 | (25.0–27.0) |
|  | ≥2 times per day | 23.5 | (22.9–24.1) | 4.4 | (3.7–5.1) | 27.9 | (27.0–28.8) |
| **High school or higher** |  |  |  |  |  |  |  |
| 0–9 | <2 times per day | 22.1 | (21.6–22.6) | 3.5 | (3.2–3.8) | 25.6 | (25.0–26.2) |
|  | ≥2 times per day | 23.8 | (23.4–24.2) | 3.5 | (3.2–3.8) | 27.3 | (26.7–27.9) |
| 10–19 | <2 times per day | 22.7 | (22.0–23.4) | 4.0 | (3.4–4.6) | 26.7 | (25.8–27.6) |
|  | ≥2 times per day | 24.5 | (23.9–24.9) | 4.0 | (3.4–4.6) | 28.5 | (27.7–29.3) |

Supplementary Table 13. DFLE, DLE, and TLE at 65 years according to the number of remaining teeth stratified by daily brushing and educational status.

Supplementary Table 14. DFLE, DLE, and TLE at 65 years according to the number of remaining teeth stratified by use of dentures and educational status.

| Number of remaining teeth | Use of dentures | DFLE | (95% CI) | DLE | (95% CI) | TLE | (95% CI) |
| --- | --- | --- | --- | --- | --- | --- | --- |
| **Men** |  |  |  |  |  |  |  |
| **Junior high school or less** |  |  |  |  |  |  |  |
| 0–9 | No | 15.8 | (16.8–17.8) | 1.3 | (1.0–1.6) | 17.1 | (16.0–18.2) |
|  | Yes | 18.7 | (18.2–19.2) | 1.4 | (1.3–1.5) | 20.1 | (19.6–20.6) |
| 10–19 | No | 18.9 | (18.1–19.7) | 1.7 | (1.4–2.0) | 20.5 | (19.5–21.5) |
|  | Yes | 19.7 | (19.1–20.3) | 1.6 | (1.4–1.8) | 21.3 | (20.6–22.0) |
| **High school or higher** |  |  |  |  |  |  |  |
| 0–9 | No | 16.7 | (15.7–17.7) | 1.1 | (0.9–1.3) | 17.8 | (16.8–18.8) |
|  | Yes | 19.6 | (19.2–20.0) | 1.3 | (1.2–1.4) | 20.9 | (20.4–21.4) |
| 10–19 | No | 19.8 | (19.0–20.6) | 1.5 | (1.2–1.8) | 21.3 | (20.4–22.2) |
|  | Yes | 20.6 | (20.1–21.1) | 1.5 | (1.3–1.7) | 22.1 | (21.5–22.7) |
| **Women** |  |  |  |  |  |  |  |
| **Junior high school or less** |  |  |  |  |  |  |  |
| 0–9 | No | 19.4 | (18.4–20.4) | 3.7 | (2.9–4.5) | 23.1 | (21.9–24.3) |
|  | Yes | 22.3 | (21.8–22.8) | 3.7 | (3.4–4.0) | 26.0 | (25.4–26.6) |
| 10–19 | No | 22.3 | (21.4–23.2) | 4.6 | (3.6–4.6) | 26.9 | (25.6–28.2) |
|  | Yes | 23.0 | (22.4–23.6) | 4.3 | (3.7–4.9) | 27.3 | (26.5–28.1) |
| **High school or higher** |  |  |  |  |  |  |  |
| 0–9 | No | 20.4 | (19.4–21.4) | 3.4 | (2.7–4.1) | 23.8 | (22.6–25.0) |
|  | Yes | 23.3 | (22.9–23.7) | 3.4 | (3.1–3.7) | 26.7 | (26.2–27.2) |
| 10–19 | No | 23.4 | (22.6–24.2) | 4.2 | (3.4–5.0) | 27.6 | (26.4–28.8) |
|  | Yes | 24.1 | (23.6–24.6) | 3.9 | (3.4–4.4) | 28.0 | (27.3–28.7) |
